# Cystic fibrosis and alpha-1 antitrypsin deficiency: case report and review of literature

**DOI:** 10.1186/s12887-022-03290-6

**Published:** 2022-05-03

**Authors:** Evi Jaspers, Ine Van Dijck, Ilse Hoffman, Noël Knops, Xavier Stéphenne, Peter Witters, Marijke Proesmans

**Affiliations:** 1 University Hospital Leuven, Herestraat 49, 3001 Leuven, Belgium; 2 University Hospital Saint-Luc, Avenue Hippocrate 10, 1200 Brussels, Belgium

**Keywords:** Cystic fibrosis, Alpha-1 antitrypsin deficiency, Case report, Infant liver transplant

## Abstract

**Background:**

This case report describes a child born with both cystic fibrosis (CF) and alpha-1 antitrypsin deficiency (A1ATD). Both are autosomal recessive inherited diseases, mainly affecting the lungs and the liver. The combination of both diseases together is rare and may lead to a fulminant disease with limited life span. To the best of our knowledge, no case has been reported of a patient born with both diseases.

**Case presentation:**

After an uneventful pregnancy, a male baby was born with meconium ileus. The suspected diagnosis of CF was confirmed based on the sweat test and genetic analysis. The child developed persisting cholestasis, too severe to be likely caused by CF alone and indicating an associated problem. The diagnosis of A1ATD was established based on clinical suspicion (persisting cholestasis), decreased serum alpha-1 antitrypsin and genetic analysis. Supportive therapy was started, however the boy evolved to rapidly progressive liver disease leading to liver failure which necessitated an infant liver transplantation.

**Conclusions:**

This case illustrates the complexity of care in case of two severe inherited diseases as well as post solid organ transplant care.

## Background

In cystic fibrosis (CF), CFTR (cystic fibrosis transmembrane conductance regulator) mutations result in defective chloride transport at the epithelial cell membrane and disturbed water and electrolyte balance leading to thick mucus and decreased mucus clearance [1, 2]. In the lung, excessive neutrophilic infiltration and an excessive proinflammatory cytokine production may be partially unrelated to bacterial infection, since it occurs even in absence of bacterial infection [1, 2]. The normal anti-neutrophil elastase protection becomes overwhelmed leading to inflammation and lung destruction [2].

The most common CF presentation in children is chronic or recurrent respiratory tract infection, associated with signs of malabsorption due to pancreatic insufficiency [2]. Other early symptoms are meconium ileus (MI) and neonatal cholestasis. MI is a congenital intestinal obstruction strongly suggestive for CF, as it occurs in 10—20% of CF patients [2].

Cystic fibrosis associated liver disease (CFLD) is present in 10—40% of patients and usually occurs around puberty. One of the risk factors for development of CFDL is MI [3]. Although the exact pathogenesis is not yet unraveled, CFTR dysfunction may lead to bile duct obstruction with inflammation and subsequent liver fibrosis in infants [4]. Liver dysfunction can present as neonatal cholestasis which can be aggravated if parenteral nutrition is needed [4]. In case of severe persisting cholestasis or liver failure, CFLD is however rarely the sole cause.

A1ATD is one of the most frequently inherited genetic disorders within the Caucasian populations, with an incidence of 1/ 1500—4500 people and caused by autosomal recessive pathogenic mutations in the SERPINA1 gene, encoding for A1AT. The latter is a serine protease inhibitor (SERPIN) mainly synthesized in hepatocytes that inhibits neutrophil proteases. Over 120 alleles identified are classified from ‘A’ to ‘Z’. The deficient Z-allele accounts for approximately 95% of A1ATD cases, 65—90% of the homozygous patients (Pi*ZZ) have pulmonary and/or hepatic symptoms. Symptoms rarely appear before 25 years of age; most infants remain asymptomatic throughout childhood [5].

We report on a boy with early CF diagnosis presenting with MI and rapidly progressive liver disease leading to liver failure and later diagnosed with alpha-1 antitrypsin deficiency (A1ATD). The incidence of having both diseases is approximately one in every 3 to 31,5 million people [1, 5].

## Case presentation

This boy was born term to non-consanguineous parents after an uneventful pregnancy. On day one, the child presented with a lower abdominal obstruction with on barium enema a microcolon and malrotation. Surgical exploration confirmed the suspicion of meconium ileus and malrotation, treated with a Bishop-Koop procedure and derotation. The postoperative course was complicated by a reintervention for an intestinal perforation with peritonitis followed by a prolonged paralytic ileus and need for ventilatory support.

A sweat test performed at the age of five days established the diagnosis of cystic fibrosis (sweat chloride by iontophoresis of 93 mmol/L). Later newborn screening results for CF came back positive with an immunoreactive trypsinogen (IRT) value of 70 ng/mL and a homozygous F508del mutation. CF treatment was started according to standards of care.

The boy developed persisting cholestasis which was initially attributed to cystic fibrosis and the prolonged need for total parenteral nutrition. In the following weeks, the cholestasis parameters and liver function test significantly worsened (see Table 1). Other causes of neonatal cholestasis such as congenital infections, endocrinological and metabolic diseases were excluded. Liver ultrasound could not demonstrate dilated bile ducts, hence excluding obstructive causes except for biliary atresia. Nevertheless, due to the reduced plasma alpha-1 antitrypsin (A1AT) (0.28 g/L (0.88 – 1.74 g/L)), genetic analysis for A1ATD was performed and confirmed a homozygous p.Glu366Lys-mutation (Z) in exon five of the SERPINA1 gene. Genetic analysis confirmed carrier status for A1ATD as well as for the F508del mutation for both parents.

**Table 1:** Lab results: deviant results indicated with *

Age	20 days	22 days	34 days	37 days
Bilirubin total (≤ 1.18 mg/dL)	3.67*	4.81*	8.45*	8.70*
Bilirubin direct (≤ 0.50 mg/dL)	3.02*	3.91*	6.74*	7.33*
Gamma GT (≤ 230 U/L)	317*	332*	436*	451*
Alkaline phosphates (122—489 U/L)	131	159	244	288
AST (≤ 37 U/L)	63*	61*	108*	115*
ALT (≤ 41 U/L)	28	29	61*	73*
PT (INR)	1.3	/	1.1	1.1

The boy was discharged and at age 2 months weight evolution slowly improved with additional nasogastric tube feeding, but cholestasis persisted.

At the age of six months an increase in icterus and hepatosplenomegaly was seen. Biochemically there were signs of liver failure with an abnormal coagulation (PT 22.5 s, INR 2.0, despite vitamin K suppletion), hypoalbuminemia (26 mg/dL), increase in cholestasis and doubling of total bilirubin to 8 mg/dL (direct 7 mg/dL). One week later the child presented with petechiae, thrombocytopenia (40.000/mL) and persistent abnormal coagulation (PT 22.7 s, INR 1.9). Two weeks later he developed ascites, with on abdominal ultrasound for the first time a hepatofugal portal flow. The child was transferred to a reference hospital for infant liver transplantation because of decompensated chronic liver disease and related portal hypertension. While on the urgent liver transplant list, he developed an intestinal obstruction, for which a laparotomy with subsequent partial ileal resection was necessary. At the age of eight months, an orthotopic liver transplant was performed.

The post-transplant course was complicated by multiple redo-abdominal interventions due to intra-abdominal bleeding and wound infection. This resulted in short bowel disease with permanent need for parenteral nutrition. One-week post-transplant he had a severe lymphocytic mediated acute organ rejection (Banff score 8/9), which was treated with high dose corticosteroids. At the age of 16 months, a surgical restoration of transit with the implementation of a gastrostomy was performed. Weaning of parenteral nutrition was successful at the age of 2 years, after receiving parenteral nutrition for most of his life.

A1AT serum level post liver transplant was normal (1.86 g/L).

## Discussion and conclusions

Both CF and A1ATD are severe autosomal recessive inherited diseases, affecting the lungs and the liver [1, 2]. CF lung disease is characterized by a large neutrophil burden with normal levels of anti-proteases, while neutrophil burden is normal in A1ATD but levels of antiproteases are decreased [1, 2].

In the reported case, the diagnosis of A1ATD was established because the severity of the liver disease could not be explained fully by CF. Risk factors for the development of CFDL include male sex, CFTR genotype, later age at diagnosis of CF, meconium ileus, severity of pulmonary disease, pancreatic insufficiency and persistent failure to thrive [3]. Moreover, the SERPINA1 Z-allele has been proven an additional risk factor for CFDL [2].

CFLD can present with neonatal cholestasis and at later ages, from asymptomatic elevation of transaminases to cirrhosis, end-stage liver disease and portal hypertension [4]. However the majority of patients have focal changes of the liver parenchyma without clinical significance.

Liver disease in our case may be predominantly determined by A1ATD with CF. Intestinal failure associated liver disease (IFALD) is a potentially contributing factor due to the intestinal failure and period of parenteral nutrition. Moreover, the need for repeated broad-spectrum antibiotics may have influence on liver disease as well.

The incidence of A1ATD lies in the same range as the incidence of cystic fibrosis within the Caucasian populations.

The associated lung disease is caused by a “loss-of-function” defect of A1AT leading to proteolytic damage to the connective tissue matrix of the lung resulting in pulmonary emphysema, chronic obstructive pulmonary disease (COPD) and/or bronchiectasis from young adulthood [5].

A1ATD associated liver disease is caused by retention of the mutant Z A1AT within the endoplasmic reticulum (ER) of hepatocytes activating a cascade of apoptotic liver cell death with compensatory hepatocellular proliferation, leading to end-organ injury. Presenting symptoms are protracted neonatal jaundice, liver enzyme elevation, hepatitis, liver cirrhosis, hepatocellular carcinoma and rarely fulminant hepatic failure. If there is an A1AT level lower than 1.1 g/L or a strong clinical suspicion, genetic analysis is needed to establish a definite diagnosis [5].

Rapidly progressive liver disease leading to liver failure is unusual in the context of CF liver disease as well as A1ATD. We hypothesize that the combination of these 2 genetic diseases together with the need for prolonged parenteral feeding caused severe liver disease. The rapid progression may have been triggered by respiratory infection.

Exogenous alpha-1-proteinase inhibitor (alpha1-PI) has been studied as CF treatment because it inhibits neutrophil elastase activity and may reduce inflammation but further studies are needed to prove efficacy [1].

Alpha-1 -PI is the only licensed and primary disease-specific treatment solely for A1AT lung disease but there are few data in children [1]. Liver transplantation is the only curative option for A1ATD liver disease [4].

Multidisciplinary tertiary care is needed to ensure adequate treatment and follow-up. However, one team should take the lead to optimize coordination of care and to facilitate the communication between parents and the hospital. In our hospital, the CF team coordinates the care with multidisciplinary follow up of the pediatric solid organ transplant team together with hepatology and gastroenterology.

To the best of our knowledge this is the first reported case of a child born with both CF and A1ATD. It is an illustration of the complexity of care of a child with two severe diseases as well as post solid organ transplant care.

## Data Availability

Not applicable.

## Abbreviations

A1AT: Alpha-1 antitrypsin
A1ATD: Alpha-1 antitrypsin deficiency
alpha1-PI: Alpha-1 proteinase inhibitor
CF: Cystic fibrosis
CFLD: Cystic fibrosis associated liver disease
CFTR: Cystic fibrosis transmembrane conductance regulator
COPD: Chronic obstructive pulmonary disease
ER: Endoplasmic reticulum
IFALD: Intestinal failure associated liver disease
IRT: Immunoreactive trypsinogen
MI: Meconium ileus
SERPIN: Serine protease inhibitor

## References

[1] McElvaney NG (2016). Alpha-1 antitrypsin therapy in cystic fibrosis and the lung disease associated with alpha-1 antitrypsin deficiency. Ann Am Thorac Soc.

[2] Castellani C, Assael BM (2017). Cystic fibrosis: a clinical view. Cell Mol Life Sci.

[3] Witters P, De Boeck K, Dupont L (2009). Non-invasive liver elastography (Fibroscan) for detection of cystic fibrosis-associated liver disease. J Cyst Fibros.

[4] Debray D, Narkewicz MR, Bodewes FAJA (2017). Cystic Fibrosis-related Liver Disease: Research Challenges and Future Perspectives. J Pediatr Gastroenterol Nutr.

[5] Lopes AP, Mineiro MA, Costa F (2018). Portuguese consensus document for the management of alpha-1-antitrypsin deficiency. Pulmonology.

